# Apathy and Executive Function in Healthy Elderly—Resting State fMRI Study

**DOI:** 10.3389/fnagi.2017.00124

**Published:** 2017-05-09

**Authors:** Toshikazu Kawagoe, Keiichi Onoda, Shuhei Yamaguchi

**Affiliations:** Department of Neurology, Shimane UniversityIzumo, Japan

**Keywords:** age-related deterioration, executive function, cognitive apathy, neuroimaging, frontostriatal network

## Abstract

Apathy is a quantitative reduction in goal-directed behaviors, having three subtypes. Despite executive deterioration in healthy aging, researchers have not investigated the “cognitive-deficit” subtype of apathy in healthy populations, which would result from executive dysfunction. We hypothesized that a relationship between apathy and executive function (EF) would be found in healthy older adults, accompanied with neural deterioration with functional dysconnectivity between the striatum and frontal region as suggested by previous studies. A total of 100 healthy adults in a health examination system database were analyzed. The present study indicates that apathy is substantially associated with executive deterioration, which can be partially ascribed to decreased functional connectivity between the frontal and ventral striatum. Despite some limitations, our findings may contribute to research on healthy psychological aging.

## Introduction

Apathy has been described as a “simultaneous decrease in the behavioral, cognitive and emotional concomitants of goal-directed behavior due to loss of motivation” (Marin, 1991). It has been identified in most types of neurodegenerative disease (Cummings and Benson, 1988; Stuss et al., 2000; Levy and Dubois, 2006; Lanctôt et al., 2017) and is an independent risk factor for other diseases (Eurelings et al., 2014). It is an important behavioral syndrome in clinical situations because it can impair recovery from a physical or neurocognitive injury and is associated with longer hospitalization and poor response to rehabilitation (Lenze et al., 2007). Because of its importance, previous studies have sought to identify the underlying mechanism of this syndrome. Levy and Dubois (2006) proposed three subtypes of disrupted processing in the apathetic condition, that is, deficits in emotional-affective, cognitive, or auto-activation function. In brief, emotional-affective problems derive from an inability to associate affective or emotional signals with ongoing and forthcoming behaviors. The cognitive apathy is a reduction in goal-directed behavior due to impairment of the cognitive functions needed to elaborate action plans. The auto-activation deficit represents the most severe form of apathy, characterized by difficulties in self-initiating actions or thought.

Apathy in healthy populations can be alternatively conceptualized as a loss of motivation. Actually, Bonnelle et al. (2016) described loss of intrinsic motivation in healthy adults as “behavioral apathy”. Studies of healthy adults have consistently demonstrated a causal role for the basal ganglia in loss of motivation (Murayama et al., 2010), which is in accord with patient studies that highlight the importance of a frontal-basal ganglia loop in pathological apathy (Levy and Dubois, 2006; Levy, 2012). According to Levy and Dubois (2006) and Levy (2012), the cognitive apathy results from impairment of executive function (EF), which, among the various types of cognitive deterioration that human beings experience (Salthouse, 1996; Park et al., 2002), may be the function most susceptible even in healthy aging (West, 1996). There should therefore be a substantial association between EF and apathy in healthy adults, specifically in older individuals. Numerous cognitive processes have been identified as aspects of EF, including task-switching, working memory, inhibition and other types of “higher order” cognitive function. This complex array of functions hinges on the prefrontal cortex as its neural basis. In fact, a study of 367 older adults found that apathy was more common than depression, dysphoria, anxiety, or mental impairment (Adams, 2001). A longitudinal study demonstrated that apathy scores are associated with aging (Brodaty et al., 2010). Thus, the present study focused on the association between EF and apathy and investigated the neural basis of apathy in a healthy older population.

In this study, the neural basis of apathy in healthy adults was explored by resting-state functional MRI (rsfMRI), which is useful in assessing brain activation related to individual characteristics, including apathetic states (Alexopoulos et al., 2013; Onoda and Yamaguchi, 2015). Brain regions underlying the cognitive apathy include the prefrontal cortex and the basal ganglia, as revealed by clinical studies of patients with neurodegenerative disease. It is particularly emphasized that the importance of the dorsal striatum in cognitive apathy (Levy and Dubois, 2006). On the other hand, the literature on motivation in healthy populations has identified a significant contribution of the striatum (Cardinal et al., 2002). Of the regions related to the dopamine system, the ventral striatum (i.e., the nucleus accumbens, NAcc) is demonstrably associated with motivation-related behavior or pathology (Carriere et al., 2014; Bonnelle et al., 2016). Resting-state functional connectivity (rsFC) between the prefrontal cortex and NAcc seems to be significantly related to apathy in depression (Alexopoulos et al., 2013). Thus, the present study investigated the rsFC of the dorsal and/or ventral striatum with the prefrontal cortex.

We analyzed data obtained from health examinations to test two hypotheses based on previous studies. (1) There should be a specific relationship between apathetic states and EF in older healthy adults because of age-related cognitive deterioration (i.e., an increasing number of people with executive dysfunction). (2) Participants with the cognitive apathy subtype should evince reduced rsFC between the frontal region and dorsal and/or ventral striatum as compared with nonapathetic participants. Scores on multiple tests of EF were integrated into a single index to address the so-called task impurity problem (Miyake et al., 2000), which refers to problems surrounding the assessment of EF using a single measure. Because the state of apathy may be associated with depression, a depressive state was assessed with a questionnaire to control for its effect. Previous studies demonstrated that apathy and depression often coexist (Levy et al., 1998) and that depression is related to broad impairment in multiple aspects of EF (Snyder, 2013). However, these two are not always correlated (Marin et al., 1993), and we previously demonstrated that rsFC can distinguish apathy from depression (Onoda and Yamaguchi, 2015). To our knowledge, this is the first study to investigate apathy and its relationship to EF by means of functional brain imaging data in healthy adults who do not have serious cognitive impairment or signs of structural brain abnormalities.

## Materials and Methods

### Participants

Participants were selected from the health examination system database of the Shimane Institute of Health Science, Izumo City, Shimane Prefecture, Japan. This system includes records of medical, neurological, neuropsychological, MRI and blood test data of patients who had undergone rsfMRI scanning and neuropsychological testing from December 2012 to September 2015. All individuals included in the present study were over 64 years old at the time of testing and had been living independently in the community without any psychiatric treatment. Potential participants were excluded if there was any suspicion of cognitive impairment or cerebral injuries or abnormalities, including severe atrophy, cerebral hemorrhage, previous cerebral infarction including silent infarction, aneurysm, severe hypoplasia, empty sella, any kind of cyst, enlarged perivascular space, or vessel malformation. At least two specialists (radiologists, neurologists and other medical doctors) confirmed these findings in addition to standard neurological examination. Individuals having a medical history of cancer, heart disease, or severe decrease in vision or hearing were also excluded, as were those with any history of cerebral disease, stroke, psychosis, or Parkinsonism. Finally, we excluded individuals if their data had any missing values. After screening, 100 participants were included in the present analysis (57 women, mean age 72.6 years, SD 3.9, range 65–79). Excluded participants were mostly younger than the inclusion criterion (*ca*. 50% of excluded individuals), had health conditions or medical history (*ca*. 20%), cerebral abnormalities including suspicion of impairment (*ca*. 20%), or missing values (*ca*. 10%). The study was conducted in accordance with the Declaration of Helsinki (1975, as revised in 2008) and the regulations of the Japanese Ministry of Health, Labour and Welfare. The medical ethics committee of Shimane University approved the study, and all participants provided written informed consent.

### Neuropsychological and Neuropsychiatric Measures and Participant Grouping

All participants underwent a Japanese-language neuropsychological assessment, which consisted of the Mini-Mental State Examination (MMSE; Folstein et al., 1975), Verbal Fluency Test (VFT; Bechtoldt et al., 1962), Frontal Assessment Battery (FAB; Dubois et al., 2000), Wisconsin Card Sorting Test (WCST; Anderson et al., 1991) and the “Kana-hiroi” test (Kaneko, 1990). These tests tap higher order cognitive function or EF. For the VFT, individuals were asked to generate names from a specified category (i.e., vegetable) in 1 min, which is a common procedure for assessing verbal fluency. For the WCST, scores were based on the number of categories achieved and the time required. In the “kana-hiroi” test, participants picked five kana letters corresponding to A, I, U, E and O (Japanese vowels) as they occurred as the person was reading and memorizing a story written in kana. The number of letters correctly recognized in 2 min was scored. The MMSE was used for rough screening of cognitive impairment, with a cutoff point of 24/25. The scores on multiple tests of EF were integrated into a single index to address the task impurity problem. In addition to these cognitive assessments, the Self-Rating Depression Scale (SDS; Zung et al., 1965) and Apathy Scale (AS; Starkstein et al., 1992) were administered to assess depression and apathy symptoms, respectively. The AS ranges from 0 to 42 and the SDS from 0 to 80. Higher SDS and AS values indicate more apathetic and depressive characteristics. For the EF index used in this study, a single integrated value was used. As previously described, scores of EF-related tests (i.e., VFT, FAB, WCST and the “kana-hiroi” test) were converted into *Z* values across the entire sample of participants, calculated by taking the average score and collapsing it into a single index (Z-EF; Kawagoe et al., 2017).

For rsFC analysis, participants were distributed into groups based on their apathy and EF scores. Those with AS scores of 16 or higher, the cutoff point for apathy on the AS (Starkstein et al., 1992), were deemed apathetic. Apathetic participants with lower than average EF scores (*Z*-scores less than zero for the entire sample of participants) were grouped as “apathy with low EF” (ApL_EF_), and apathetic participants with higher than average EF scores were deemed “apathy with high EF” (ApH_EF_). Participants with AS scores of 15 or lower were considered “nonapathetic” (NAp).

### fMRI Data Acquisition, Preprocessing and Connectivity Analysis

Imaging data were acquired using a Siemens AG 1.5T scanner (Symphony). Twenty-seven slices parallel to the plane connecting the anterior and posterior commissures were measured using a T2*-weighted gradient-echo spiral pulse sequence, with repetition time 2000 ms, echo time 30 ms, flip angle 90°, interleave order, matrix size 64 × 64, field of view 256 × 256 mm^2^, isotropic spatial resolution 4 mm, 27 slices with 4.5 thickness and no gap. All participants underwent a 5-min rsfMRI scan after we instructed them to remain awake with their eyes closed. After the functional scan, T1-weighted images of the entire brain were obtained (192 slices, repetition time 2170 ms, echo time 3.93 ms, inversion time 1100 ms, flip angle 15°, matrix size 256 × 256, field of view 256 × 256 mm^2^, isotropic spatial resolution 1 mm).

We used Statistical Parametric Mapping software (SPM12^1^) implemented in MATLAB (MathWorks, Natick, MA, USA) for spatial preprocessing. The initial five functional volumes were deleted for magnetic field stimulation. The remaining images were realigned to remove any artifacts from head movement and corrected for differences in image acquisition time between the slices. The realigned images were normalized to a Montreal Neurological Institute (MNI) template standard space and resliced with a 3 × 3 × 3 mm voxel size. Spatial smoothing was then applied with the full width at half maximum (FWHM) equal to 8 mm. After spatial preprocessing, temporal preprocessing was conducted with the Functional Connectivity Toolbox (CONN^2^). The head movement time series, white matter signal and cerebral spinal fluid signal were regressed out from each voxel. These data were smoothed with a bandpass filter (0.01–0.08 Hz). We defined two regions of interest (ROIs), the head of the caudate nucleus of dorsal striatum and the NAcc of the ventral striatum, both bilaterally. Spherical ROIs (radius 6 mm) for the head of the bilateral caudate were defined based on an earlier meta-analysis of caudate functional connectivity (Robinson et al., 2012); it was centered at *x* = −15, *y* = 9, *z* = 19 for the left structure and at *x* = 15, *y* = 8, *z* = 21 for the right structure. For the NAcc, ROIs were created based on anatomical coordinates defined in a stereotactic investigation of the human NAcc (Neto et al., 2008). Spherical ROIs (radius 6 mm) were centered at MNI coordinates *x* = −9, *y* = 6, *z* = −4 for the left structure and *x* = 9, *y* = 6, *z* = −4 for the right structure.

### Statistical Analysis

Analysis of variance (ANOVA) and multivariate comparisons with Bonferroni tests were conducted. Chi-square was used as a test of independence for residual analyses. Pearson’s correlation coefficients were calculated to assess relationships among variables. A *p* level of 0.05 was accepted as statistically significant. Statistical analysis was performed using the SPSS software package (version 22, IBM Corp., Armonk, NY, USA). For functional connectivity matrices, Pearson correlations for the entire time course were computed against each voxel for the whole brain and transformed into *Z*-scores with Fisher’s transformation for all participants (seed-to-voxel analysis). The strength of rsFC with each ROI was compared with a one-way ANOVA model with one between-group factor. The images were thresholded using clusters determined by *Z-scores* >2.3 and a familywise error (FWE) corrected cluster significance threshold of *p* < 0.05. We performed correlation and functional connectivity analyses controlling for confounding factors, including age, sex, education and SDS, which showed significant partial correlations with the variables of interest.

## Results

### Neuropsychological and Neuropsychiatric Measures

**Figure 1:**
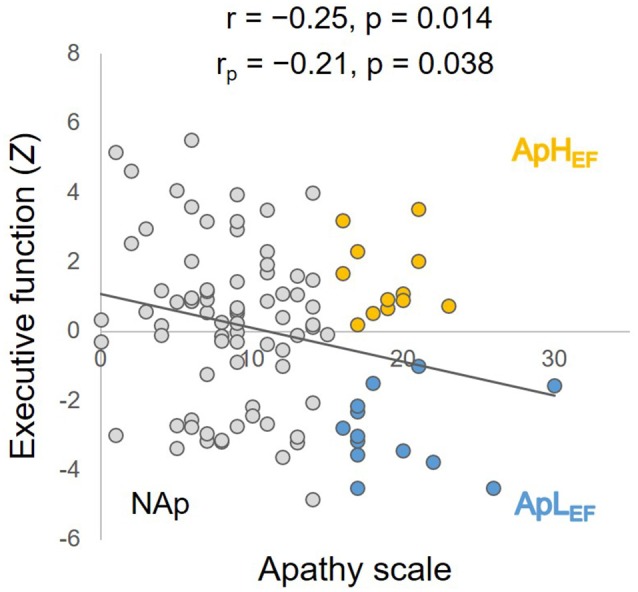
**Correlation between executive function (EF) and apathy, with simple and partial coefficients and statistical *p* values**. Orange circles represent ApH_EF_ data, blue circles ApL_EF_ data and gray circles NAp data. ApL_EF_: apathy with low EF; ApH_EF_: apathy with high EF; NAp: nonapathetic.

The measured data from the overall sample are listed in the rightmost column of Table 1. The significant correlation between age and Z-EF (see “Materials and Methods” Section) in the entire sample (*r* = −0.34, *p* < 0.001) was consistent with earlier findings and supported the present hypothesis. Age itself was not significantly related to AS (*r* = 0.03, *p* = 0.714). The correlation between AS and EF calculated for the entire sample was significant (*r* = −0.25, *p* = 0.014; Figure 1), whereas EF and SDS were not significantly correlated in this study (*r* = −0.04, *p* = 0.70). The correlation between AS and EF remained significant after considering possible confounding factors such as age, sex, education and SDS (*r*_p_ = −0.21, *p* = 0.038), although AS and SDS (*r* = 0.50, *p* < 0.001) were significantly correlated.

**Table 1:** **Demographics and measured data for each group**.

Variables	ApL_EF_	ApH_EF_	NAp	All
Age (SD)	72.6 (3.8)	72.3 (3.7)	72.4 (4.1)	72.6 (3.9)
N (women)	13 (9)^a^	12 (9)^a^	75 (38)	100 (57)
Education (SD)	11.3 (2.9)	12.1 (2.6)	13.6 (2.7)	13.1 (2.8)
MMSE (SD)	27.8 (2.3)	28.9 (1.3)	28.6 (1.6)	28.5 (1.6)
SDS (SD)	36.3 (6.3)	42.6 (4.7)^b^	32.9 (6.9)	34.5 (7.3)
AS (SD)	19.6 (4.2)	18.9 (2.2)	8.4 (3.7)	11.1 (5.9)
Z-EF (SD)	−2.8 (1.1)	1.47 (1.1)	0.3 (2.3)	−4.7^−16^(2.3)

*Note: ^a^Sex ratio is statistically different from expected value. ^b^Significant difference from ApL_EF_. Group based on appropriate post hoc tests. ApL_EF_, apathy with low executive function; ApH_EF_, apathy with high executive function; NAp, nonapathetic; MMSE, mini-mental state examination; SDS, self-rating depression scale; AS, apathy scale; Z-EF, *Z*-transformed scores of executive function*.

We then compared participants in three age groups. Table 1 displays demographics and measurements of each age group, along with results of statistical tests. We conducted a chi-square test on sex ratios and between-subject one-way ANOVAs on the remaining data. Sex ratios were significantly different between these groups (*χ*^2^ = 9.56, *p* = 0.008, Cramer’s *V* = 0.44). Residual analyses showed significantly different sex ratios for ApL_EF_ (absolute adjusted residual = 3.4, *p* < 0.01) and ApH_EF_ groups (absolute adjusted residual = 2.6, *p* < 0.01), indicating that sex factor is substantially affect the relationship between EF and apathy (Table 1). ANOVAs revealed a significant group difference only for SDS (*F*_(2,97)_ = 3.88, *p* = 0.024, *η*^2^ = 0.01). *Post hoc* tests with Bonferroni correction indicated a significant difference only between ApL_EF_ and ApH_EF_ groups (*p* = 0.020; all other differences, *p*s > 0.1). We also conducted further analyses to demonstrate that the participants were not out of the normal range for ApL_EF_. The mean FAB and SDS scores in the ApL_EF_ group were 14.9 and 42.6, respectively, which were not considered abnormal based on normative data (Fukuda and Kobayashi, 1973; Dubois et al., 2000).

### fMRI Measures

**Figure 2:**
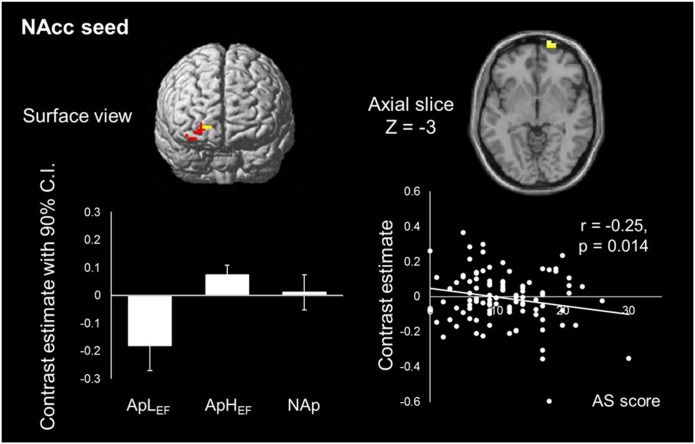
**Map of NAcc resting-state functional connectivity (rsFC) and its correlation with the index of apathy in the entire sample**. Only one cluster is significantly different between groups. ApL_EF_: apathy with low EF; ApH_EF_: apathy with high EF; NAp: nonapathetic; NAcc: nucleus accumbens.

To test our hypothesis of reduced rsFC between the frontal area and caudate and/or NAcc ROIs, seed-based analyses were conducted. There was no surviving voxel when the caudate was the seed, indicating that the connection to the caudate head was not significantly different between the three groups (ApL_EF_, ApH_EF_ and NAp). At the same time, rsFC between the NAcc and right frontal pole differed significantly between the groups (Figure 2). Detailed analysis of this difference showed that low rsFC was observed in the ApL_EF_ group. Its coordinate was *x* = 21, *y* = 72, *z* = −3 with *Z*-score = 3.87 and *k* = 18 (*p* < 0.05, FWE corrected). No other region survived at the given threshold. rsFC strength was marginally correlated with AS (*r* = 0.20, *p* = 0.049), as plotted in Figure 2.

## Discussion

We hypothesized that a relationship would exist between apathy and EF in healthy older adults, and that apathetic participants with lower EF would present with lower rsFC from the dorsal and/or ventral striatum to the frontal region. Results indicated that even in healthy older adults, apathy was associated with deterioration of EF, and rsFC between the ventral striatum and frontal region was degraded.

In the entire sample of older adults, there was a significant correlation between apathy and EF, which remained significant after controlling for age, sex, education and SDS. This could have been caused by the coupling between aging and frontal executive deterioration (Salthouse, 1996; West, 1996; Park et al., 2002). Depression, which can coexist with apathy (Marin et al., 1993; Levy et al., 1998), and executive dysfunction (Snyder, 2013) were substantially correlated with apathy (*r* = 0.50) in the present data set. While there was a significant relationship between EF and apathy, depression was not associated with EF. The lack of an association between EF and depression, which is incompatible with a previous meta-analysis (Snyder, 2013), might be due to the study population, as a level of depression within the normal range may not be related to executive dysfunction. To date, correlation between EF and apathy has mainly been reported in patients with known disorders (McPherson et al., 2002; Esposito et al., 2010). Cognitive decline, particularly in EF, could be related to apathy in healthy older adults who are independently living without general cognitive loss or any signs of structural brain abnormalities. This coincides with the cognitive apathy observed in patients (Levy and Dubois, 2006; Levy, 2012).

In the present study, we demonstrated specific degradation of rsFC between the NAcc and frontal region in apathetic older adults with lower EF. This connectivity may play a causal role in apathy in healthy older adults. This possibility is supported by the significant correlation between the rsFC of this network and AS in our overall sample. The region in which the present association was found was the frontal pole, a region of the anterior part of prefrontal cortex. The function of this area, which is not yet well understood, is high-order cognitive control, such as integrating the outcomes of two or more separate cognitive operations for a higher behavioral goal (Ramnani and Owen, 2004) and maintaining a task’s main goal while achieving concurrent subgoals (Koechlin et al., 1999). Previous studies indicated that this region provides access to reward-related information and is involved in preparation and execution of reward-motivated behavior (Pochon et al., 2002). These studies suggest that (extrinsic) motivation is processed by the frontal pole to achieve behavioral preparation and execution. The rsFC between the NAcc and prefrontal area implies the deterioration of such motivational processing in healthy older adults, which is in accord with previous findings on pathologic apathy (Alexopoulos et al., 2013). The ventral striatum is a core component of dopaminergic pathways. Thus, dopamine-dependent apathy is likely to occur with dysfunction in striatal dopamine and frontal-basal ganglia circuits (Levy, 2012). Meanwhile, when the caudate was seeded, no areas showing a significant difference between groups were depicted, although the rsFC between the dorsal striatum and frontal region represents a key network underlying the cognitive apathy in clinical patients (Levy and Dubois, 2006). Considering the present neuroimaging results, it is possible that the apathy assessed by AS in this healthy older population is qualitatively different from that in patients with diagnosed neurologic dysfunction. Apathy in healthy older adults may be related more to deterioration in the motivation process supported by the network between the NAcc and anterior prefrontal cortex than to a pathologic problem of behavioral execution.

We clearly showed a close relationship between EF and apathy in healthy older adults, with evidence of altered functional connectivity associated with aging. However, some limitations should be noted. Although the participants in this study were healthy at the time of investigation, 25% were deemed to have apathy according to their AS scores (Starkstein et al., 1992). This proportion of apathetic individuals was greater than that observed in a prior study on older adults (Brodaty et al., 2010) using the Apathy Evaluation Scale. This scale provides a unidimensional apathy measure that is similar to the SDS; thus, it may be useful to employ different types of assessments in future studies. For example, a multidimensional scale was recently developed to assess subtypes of the Levy and Dubois’s classification (Radakovic and Abrahams, 2014). Such assessment tools focus on differences in the pathophysiology of apathy across neurocognitive conditions. Another limitation is that we applied the median split for Z-EF to define the high and low EF group, although a *Z*-score less than 0 on the EF composite does not necessarily equal low EF. This was a rather arbitrary choice mainly intended to magnify the statistical power in assessing group differences. A larger sample size is needed to further evaluate the biological evidence described above.

Apathy is not only a result of neurodegenerative diseases (Cummings and Benson, 1988; Stuss et al., 2000; Levy and Dubois, 2006). Thus, our findings may contribute to research on healthy physical aging. The present results also suggest that the underlying neurobiology of apathy is not common across neurocognitive conditions (Lanctôt et al., 2017), and additional investigation is required to uncover the mechanisms of apathy in the healthy population.

## Author Contributions

TK: study concept and design, data analysis and interpretation, manuscript preparation. KO: data analysis, critical review of manuscript. SY: critical review of manuscript.

## Conflict of Interest Statement

The authors declare that the research was conducted in the absence of any commercial or financial relationships that could be construed as a potential conflict of interest.

## Footnotes

^1^http://www.fil.ion.ucl.ac.uk/spm

^2^http://www.alfnie.com/software
